# Attenuated succinate accumulation relieves neuronal injury induced by hypoxia in neonatal mice

**DOI:** 10.1038/s41420-022-00940-7

**Published:** 2022-03-28

**Authors:** Mengdi Zhang, Yao Cheng, Yujie Zhai, Yaru Cui, Wenshen Zhang, Hongwei Sun, Wenyu Xin, Ling Zhou, Xue Gao, Shucui Li, Hongliu Sun

**Affiliations:** 1 School of Pharmaceutical Sciences, Binzhou Medical University, 264003 Yantai, China; 2 The Sixth Scientific Research Department, Shandong Institute of Nonmetallic Materials, 250031 Jinan, China

**Keywords:** Neurological disorders, Diseases of the nervous system

## Abstract

Hypoxia causes neonatal neuronal damage. However, the underlying mechanism remains unclear. This study aimed to explore the changes in succinate levels and identify the mechanisms underlying their contribution to hypoxia-induced damage in newborn mice. The neonatal C57BL/6J mouse hypoxia model was used in our study. We evaluated the levels of succinate, iron, reactive oxygen species (ROS), and mitochondrial ROS, and assessed mitophagy, neuronal damage, and learning and memory function, after hypoxia treatment. The neonatal mice showed increased succinate levels in the early hypoxia stage, followed by increased levels of oxidative stress, iron stress, neuronal damage, and cognitive deficits. Succinate levels were significantly reduced following treatment with inhibitors of succinate dehydrogenase (SDH), purine nucleotide cycle (PNC), and malate/aspartate shuttle (MAS), with the corresponding attenuation of oxidative stress, iron stress, neuronal damage, and cognitive impairment. Reversal catalysis of SDH through fumarate from the PNC and MAS pathways might be involved in hypoxia-induced succinate accumulation. Succinate accumulation in the early period after hypoxia may crucially contribute to oxidative and iron stress. Relieving succinate accumulation at the early hypoxia stage could prevent neuronal damage and cognitive impairment in neonatal hypoxia.

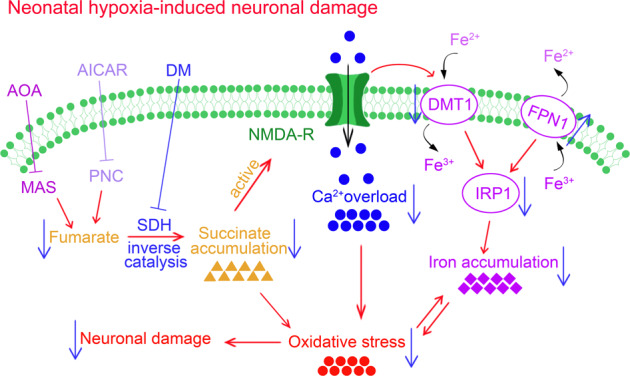

## Introduction

Hypoxia is the main cause of hypoxic-ischemic encephalopathy. In the neonatal period, hypoxia may cause neuronal damage, brain dysfunction, epilepsy, impaired consciousness, and learning and memory deficits [1, 2]. Therefore, there is a need to identify the potential mechanisms and strategies for preventing neonatal hypoxia-induced brain damage.

Neonatal hypoxia leads to active oxygen accumulation [3]. The brains of newborns are vulnerable to oxidative stress, which is a critical contributor to brain damage [4]. Reactive oxygen species (ROS) and mitochondrial ROS (mito-ROS) could cause neuronal damage [2, 5, 6]. For example, in neonatal hypoxic-ischemic encephalopathy, excitatory amino acids, including glutamate and aspartic acid, activate N-methyl-D-aspartate (NMDA) receptors to extensively produce ROS and mito-ROS, which causes hypoxic-ischemic brain damage [7]. Moreover, severe oxidative stress, mitochondrial dysfunction, and neuronal damage have been observed in overexcitation-induced epilepsy and seizures [8, 9].

After perinatal hypoxia, the neonatal brain undergoes neurotoxic events that are closely related to ROS production, including glutamate release and NMDA receptor activation [10, 11]. Overexcitation resulting from NMDA receptor activation causes mitochondrial dysfunction [12]. Mitochondria are the main source of intracellular ROS [13]. ROS are mainly produced by mitochondrial defects [14, 15], which was first observed in patients with hereditary mitochondrial disease and epilepsy [16]. A subsequent study showed that hypoxia partially inhibits mitochondrial electron transport and generates a redox reaction in the electron carrier, and therefore increases ROS production [17].

Succinate accumulation, a critical contributor to the Krebs cycle and glutamate-GABA shunt [18], is closely associated with NMDA receptor activation and oxidative injury. Succinate accumulation induces mitochondrial ROS production, oxidative stress, and neuronal damage [19]. Moreover, succinate has excitatory effects. Succinate treatment causes convulsions in mice [20]. NMDA receptors are involved in oxidative damage caused by succinate accumulation [21]. MK-801 (an NMDA receptor antagonist) attenuates convulsions and oxidative damage caused by succinate accumulation [21]. Hypoxia causes glutamate release and NMDA receptor activation [22]. Further, excessive succinate levels have been observed in the cerebral cortex of rats after hypoxia [23]. Therefore, succinate accumulation might participate in hypoxia-induced oxidative stress and neuronal injury by overexcitation.

Iron accumulation is closely associated with oxidative stress and NMDA receptor activation, all of which contribute to neuronal damage [24]. Hypoxia exposes the developing brain of newborns to oxidative stress [25]. Iron regulatory protein 1 (IRP1) acts as an ROS sensor. Excessive ROS levels activate IRP1 that results in increased expression of the transferrin receptor (TfR) and divalent metal transporter 1 (DMT1). These promote iron uptake under conditions of iron deficiency, eventually resulting in increased iron levels [26, 27]. Additionally, the nervous system is rich in free iron, which gets exposed to the accumulated H_2_O_2_ produced because of hypoxia. Numerous highly reactive hydroxyl radicals are generated through the Fenton reaction, which destroy lipids and DNA [28], and therefore increases cytotoxicity [3]. Additionally, perinatal hypoxia can trigger glutamate release, NMDA receptor activation, and energy failure [10, 11]. Furthermore, glutamate-induced excitotoxicity disturbs the iron metabolism system, including iron ion transporters, exporters, and storage proteins [29]. Activated NMDA receptors upregulate DMT1 expression and increase iron inflow, which causes excessive iron accumulation. This leads to superfluous ROS production through the Fenton reaction [28]. Accordingly, there is an established promoting effect between excessive ROS and iron.

Taken together, iron stress and oxidative stress may be involved in succinate accumulation in neonatal hypoxia-induced neuronal damage. Accordingly, the purpose of our study is to explore the possible roles, mechanisms, and sources of succinate accumulation in hypoxia-induced neuronal injury in neonates.

## Materials and methods

### Animals

C57BL/6J mice (Certificate No. 20190003; Pengyue Experimental Animal Co. Ltd, Jinan, China) at 20 days gestation were placed in a separate cage in 12-h light/dark cycles at constant temperature (22 ± 1 °C), with water and food *ad libitum*. Experiments were conducted on postnatal day 7 (P7) in C57BL/6J pups. Experiments were conducted according to the ethical guidelines of the Binzhou Medical University Animal Experimentation Committee (approval no. 2019012), the Helsinki Declaration of 1975, and the National Institutes of Health Guide for the Care and Use of Laboratory Animals (NIH Publications No. 8023, revised 1996). All relevant experiments minimized the number and suffering of the animals. In total 484 C57BL/6J mice were used. During the experiment, the data acquisition and analysis were blinded.

### Hypoxia-induced seizures

P7 pups from the same litter were randomly divided into control, hypoxia, and intervention groups. Pups weighing less than 2 g were excluded. Hypoxia treatment of P7 pups was performed as previously described [30–32].

As previously described, 5% oxygen/95% nitrogen (Rulin Gas Ltd., China) [32, 33] was used in the hypoxic group (*n* = 10) for 15 min. The control group was exposed to 21% oxygen/79% nitrogen (*n* = 10) for 15 min.

### Pharmacological manipulations

According to the previous reports [19], the competitive succinate dehydrogenase (SDH) inhibitor, dimethylmalonate (DM; CAS, 136441-250 G; 1.15 g/ml dissolved in saline, 1 ml/kg; Sigma-Aldrich, USA), was intraperitoneally injected 15 min before hypoxic treatment in the hypoxia + DM group (*n* = 10). Additionally, the hypoxia + saline group was treated using saline before hypoxia (*n* = 10). As an inhibitor of the malate/aspartate shuttle (MAS), aminooxyacetate acid (AOA; CAS, 15R0027H; 6.25 μg/μl in saline, 1 ml/kg; J&W Pharmlab, Shanghai, China) was microinjected into the lateral ventricle 30 min before hypoxia treatment (hypoxia + AOA group, *n* = 12); the hypoxia + saline group was treated with saline (*n* = 10). Additionally, 5-aminoimidazole-4-carbox-amide-1-b-D-ribofuranoside (AICAR; CAS, 01177254; 0.3 μg/μl in saline, 1 ml/kg; Acros, Belgium), a specific inhibitor of the purine nucleotide cycle (PNC), was microinjected into the lateral ventricle 60 min before hypoxic inhalation in the hypoxia + AICAR treatment group (*n* = 8); the hypoxia + saline group received saline (*n* = 8).

### Succinate detection

Five mice from each group underwent anesthesia and brain extraction at 5 min, 30 min, and 1 h after hypoxia treatment. The cortex and hippocampus were separated and used for succinate colorimetry (MAK184, Sigma-Aldrich, USA) to measure succinate levels, as previously described [19].

### Dichlorodihydrofluorescein (DCF) assay

As previously described [19], the cortex and hippocampus were obtained from five mice at 30 min, 24 h, 3 days, and 2 months after hypoxia [32], followed by preparation of a 250-μl single-cell suspension. The cells were incubated with DCFH-DA (P0011, Beyotime Institute of Biotechnology, Shanghai, China). Fluorescence intensity was detected by a fluorescence microplate reader (Thermo, USA), with excitation and emission wavelengths of 488 nm and 525 nm, respectively.

### Determination of mitochondrial ROS

As previously described [2], the cortex and hippocampus were obtained from five mice per group at 30 min, 24 h, 3 days, and 2 months after hypoxia, respectively, followed by preparation of a 250 μl single-cell suspension. Next, 1 ml mito-SOX working solution (M36008, 5 μM, Thermo Fisher, USA) was added to the single-cell suspension. Measurements were performed at excitation and emission wavelengths of 510 nm and 580 nm, respectively, with a flow cytometer (Becton, Dickinson and Company, USA) and fluorescence microplate reader (Thermo, USA), respectively.

### Iron content detection

As previously described [2], the hippocampus and cortex were obtained from five mice at 30 min, 24 h, 3 days, and 2 months after hypoxia. Subsequently, the iron content in the tissues was measured using the iron content detection kit (DIFE008, Bioassay Systems, USA).

### Learning and memory ability

Morris water maze test was used to assess spatial learning and memory 1.5 months after hypoxia treatment (ZS-001, Zhongshi Di Chuang, Beijing, China) [34]. Details regarding the Morris water maze test have been previously described [2]. In this test, we measured the latency to finding the platform, the cumulative number of passing over the platform, the target percentage, and opposite quadrant time [35, 36].

### Fluoro-Jade B (FJB) staining

FJB staining was used to evaluate neuron injury [37]. As previously described [2], the FJB kit (AG310, Millipore, Burlington, MA, USA) was used to detect neuronal damage in five mice per group. The positive signals were manually counted under a non-fluorescent microscope [38].

### Western blot analysis

As previously described [2], at 24 h, 3 days, and 2 months after hypoxia, we extracted proteins with a commercial extraction reagent kit (Beyotime Institute of Biotechnology, China). Anti-rabbit FPN1 (1:2000, ab58695, UK), IRP1 (1:1000, ab236773, Abcam, UK), LC3B (1:2000; ab48394, Abcam, UK), caspase-3 (1:1000, 9662, Cell Signaling Technology, USA), activated caspase-3 (1:1000, ab2302, Abcam, UK), and glyceraldehyde 3-phosphate dehydrogenase (GAPDH; 1:3000; AB-P-R001, Kangcheng, Zhejiang, China) antibodies were used for western blotting analysis, based on chemiluminescent luminescent image analysis (ImageQuant LAS 500, USA). Grayscale analysis was performed on the target band using Image J (version 1.37, National Institutes of Health, Bethesda, MD), and the results are expressed as a ratio of the optical density of the target protein band to that of GAPDH.

### Immunohistochemistry

As previously described [2], tissue slices obtained at 24 h, 3 days, and 2 months from each group underwent immunohistochemical staining with IRP1/DMT1/DAPI and LC3B/TOMM20/DAPI. The primary antibody mixture included anti-rabbit IRP1 (1:200, ab236773, Abcam, UK), LC3B (1:200, ab48394, Abcam, UK), anti-mouse TOMM20 (1:200; ab56783, Abcam, UK), and DMT1 (1:200, ab55735, Abcam, UK). Images were observed with a fluorescence microscope (Olympus, Japan), and quantified with ImageJ V.1.37 software (National Institutes of Health, Bethesda, MD, USA).

### Electron microscopy

As previously described [2], two mice were randomly selected for cardiac perfusion. Further, the entorhinal cortex (EC) and dentate gyrus (DG) in these mice were obtained 2 months after hypoxia treatment. Uranyl acetate and lead citrate solution were used for double staining. Images were obtained with a transmission electron microscope (ZEISS, Germany).

### Statistical analyses

Data are presented as mean ± SD. Statistical tests were justified for every figure, and the data met normal distribution and variance homogeneity. Statistical comparisons were carried out with SPSS (version 25.0; SPSS Inc., USA). Based on the data obtained from the pre-experiment, the sample size was estimated using a balanced one-way ANOVA. The platform latency in the Morris water maze was analyzed by two-way analysis of variance (ANOVA), and other data were analyzed by one-way ANOVA with Dunnett’s T3 post-hoc test. *P* < 0.05 was considered statistically significant.

## Results

### Increased levels of succinate, DCF, mito-SOX, and iron after hypoxia treatment

**Fig. 1:**
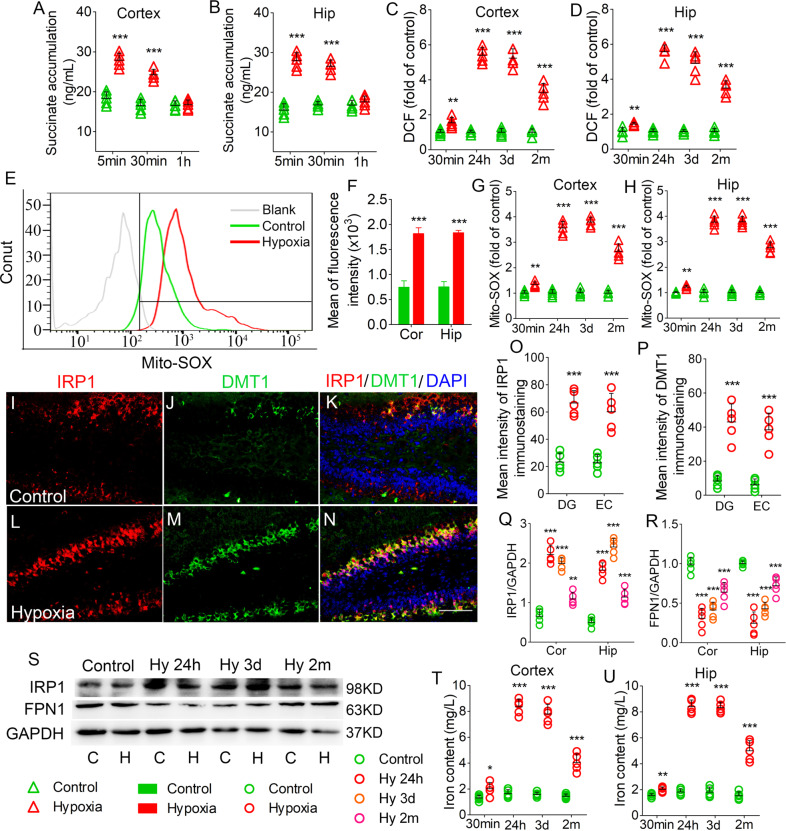
Increased levels of succinate, DCF, mito-SOX, and iron after hypoxia treatment. Increased levels of succinate (**A**, **B**), DCF level (**C**, **D**), and mito-SOX (**G**, **H**) in the hippocampus and cortex in hypoxia-induced seizure. **E**, **F** Flow cytometry-based quantification of hippocampal mito-SOX level. **I**–**N** Increased immunoreactivity of IRP1 (red), DMT1 (green) in the DG region after hypoxia. Blue, DAPI. **O**, **P** Quantified changes of IRP1 and DMT1 in the EC and DG. Bar = 50 μm. **Q**–**S** Levels of IRP1 and FPN1 in the hippocampus and the cortex estimated using western blotting. *n* = 5/group. **T**, **U** Levels of iron (*n* = 5 per time point). *P < 0.05, **P < 0.01, and ***P < 0.001, all compared with controls (One-way ANOVA).

Succinate levels increased significantly in the cortex and hippocampus at 5 min and 30 min after hypoxia administration (Fig. 1A, B). However, compared with control, there was no significant difference at 1 h (cortex, *P* = 0.803, Fig. 1A; hippocampus, *P* = 0.350, Fig. 1B). Moreover, DCF levels were increased significantly after 30 min of hypoxia administration (cortex, *P* = 0.007, Fig. 1C; hippocampus, *P* = 0.004, Fig. 1D), as well as at 24 h, 3 days, and 2 months (Fig. 1C, D). Mito-SOX levels showed similar changes to DCF levels (Fig. 1G, H). Figure 1E, F present the mito-SOX levels detected by flow cytometry.

Moreover, we evaluated the levels of iron and iron-related proteins after hypoxia treatment. Immunohistochemistry analysis revealed increased fluorescence intensity of DMT1 and IRP1 in the hippocampus, EC, and piriform cortex (PC) from 24 h onwards. Figure 1I–P shows changes in the DG and EC at 3 days after hypoxia. Western blot analysis revealed increased IRP1 levels (Fig. 1Q, S) and decreased FPN1 levels (Fig. 1R, S) in the cortex and hippocampus from 24 h after hypoxia treatment. Moreover, there were significantly increased iron levels in the cortex and hippocampus from 30 min after hypoxia treatment (Fig. 1T, U).

### Increased mitophagy and neuronal injury after hypoxia treatment

**Fig. 2:**
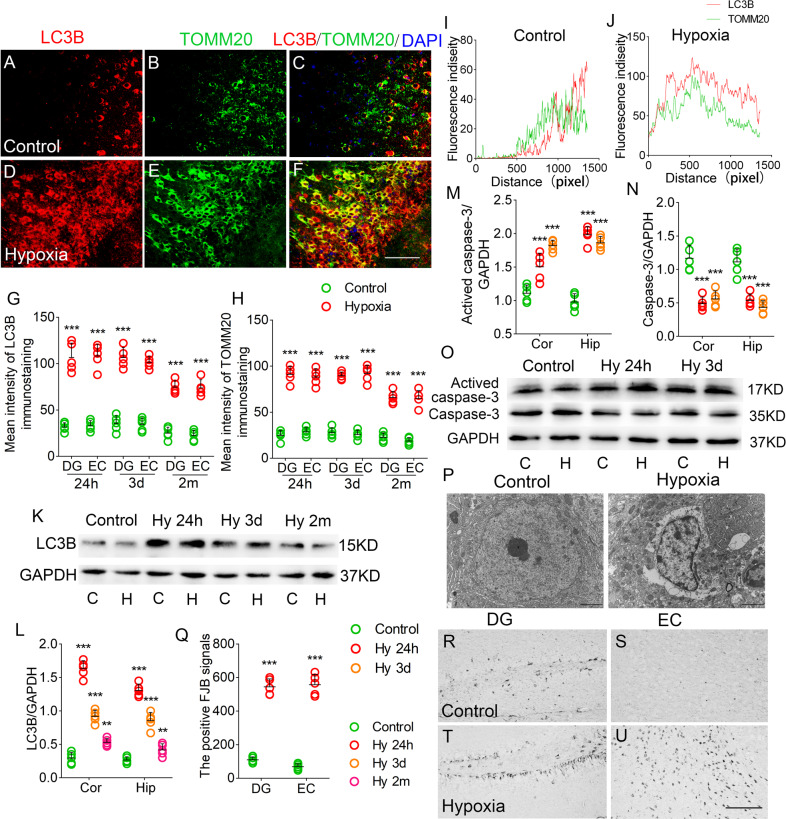
Increased mitophagy and neuronal injury after hypoxia treatment. **A**–**F** Increased immunoreactivity of LC3B (red) and TOMM20 (green) in the EC region after hypoxia. Blue, DAPI. **G**, **H** Quantification of LC3B and TOMM20 fluorescence intensity in DG and EC. Bar = 50 μm. **I**, **J** Plots of pixel intensity of LC3B and TOMM20. The levels of LC3B (**K**, **L**), activated caspase-3 and caspase-3 (**M**–**O**) in the hippocampus and cortex detected by western blotting. **P** Neurons in the DG observed with transmission electron microscopy. **Q**–**U** Positive FJB signals in DG and EC in each group after hypoxia. Bar = 50 μm, *n* = 5/group. **P < 0.01, and ***P < 0.001, all compared with controls (One-way ANOVA).

There was significantly increased fluorescence intensity of LC3B/TOMM20 in the hippocampus, EC, and PC from 24 h after hypoxia treatment (e.g., EC, *P* < 0.001, Fig. 2A–J; DG, *P* < 0.001, Fig. 2G, H). Moreover, the most positive LC3B signals overlapped with TOMM20, which is the mitochondrial marker [39] (Fig. 2A–F). The spatial and temporal overlaps of LC3B and TOMM20 have been preliminary analyzed (Fig. 2I, J). The results indicate that mitophagy might be the chief form of increased autophagy. Western blotting also revealed increased LC3B levels in the hippocampus and cortex after hypoxia treatment (Fig. 2K, L).

Western blotting was used to evaluate hypoxia-induced changes in apoptosis. There were increased levels of activated caspase-3 (Fig. 2M, O), and reduced levels of caspase-3 (Fig. 2N, O) from 24 h after hypoxia treatment. Moreover, there was significant neuronal damage in the hippocampus, EC, and PC using FJB staining (e.g., DG, *P* < 0.001; EC, *P* < 0.001; Fig. 2Q–U). Figure 2P presents representative changes in neuronal damage detected by transmission electron microscopy.

### DM treatment reversed succinate accumulation, oxidative stress, and iron stress induced by hypoxia treatment

**Fig. 3:**
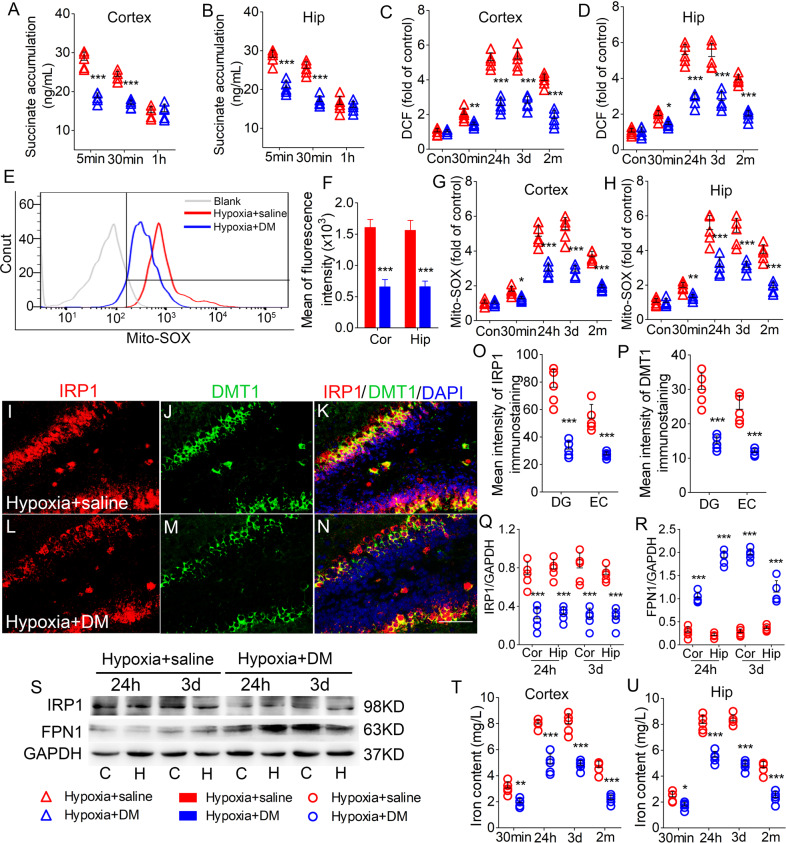
DM reversed the succinate accumulation, oxidative stress, and iron stress induced by hypoxia treatment. Decreased levels of succinate (**A**, **B**), DCF (**C**, **D**), and mito-SOX (**G**, **H**) in the hippocampus and cortex in DM-treated mice. **E**, **F** Flow cytometry-based quantification of hippocampal mito-SOX level. **I**–**N** Decreased immunoreactivity of IRP1 (red) and DMT1 (green) in the DG region after DM treatment. Blue, DAPI. **O**, **P** Changed levels of IRP1 and DMT1 in the EC and DG. Bar = 50 μm. **Q**–**S** Expression of IRP1 and FPN1 in the hippocampus and cortex estimated using western blotting. *n* = 5/group. **T**, **U** Levels of iron (*n* = 5 per time point). *P < 0.05, **P < 0.01, and ***P < 0.001, all compared with hypoxia + saline group (One-way ANOVA).

DM, which is an SDH inhibitor, was used to evaluate the possible roles and sources of succinate accumulation. Succinate levels were reduced (Fig. 3A, B), with accompanying decreases in DCF (Fig. 3C, D) and mito-SOX levels (Fig. 3E–H), in the cortex and hippocampus of DM-treated mice compared with saline-treated controls.

The results of immunohistochemistry suggested that DM intervention significantly reduced IRP1 (e.g., DG, *P* < 0.001, Fig. 3I, L,O; EC, *P* < 0.001, Fig. 3O) and DMT1 levels (Fig. 3J, M, P) in the cortex and hippocampus at 3 days after hypoxia. A similar reduction was found at the 24 h time point (data not shown). Western blotting analysis also revealed decreased IRP1 levels in the cortex and hippocampus (Fig. 3S, Q), with accompanying increased FPN1 levels (Fig. 3S, R) after DM treatment. Moreover, the increased iron levels from 30 min after hypoxia treatment were significantly reduced in DM intervention mice (Fig. 3T, U).

### DM reversed the increased mitophagy, neuronal damage, and cognitive deficits induced by hypoxia treatment

**Fig. 4:**
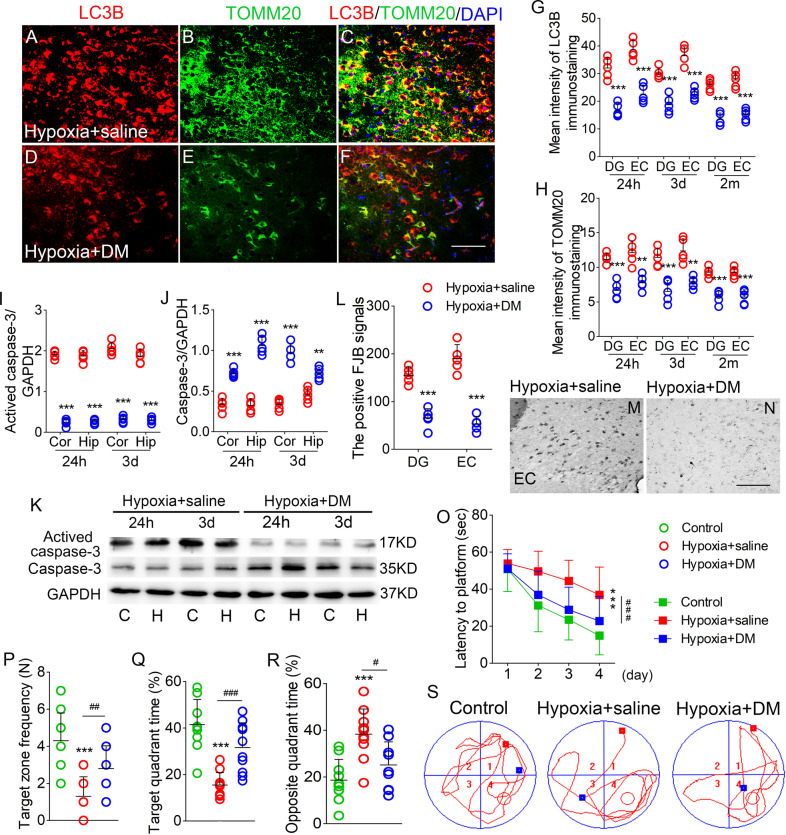
DM reversed the increased mitophagy, neuronal damage, cognitive deficits induced by hypoxia treatment. **A**–**F** Decreased immunoreactivity of LC3B (red), TOMM20 (green) in the EC after DM treatment. Blue, DAPI. **G**, **H** Fluorescence intensity of LC3B and TOMM20 in DG and EC. **I**–**K** The expression of activated caspase-3 and caspase-3 in the cortex and hippocampus detected by western blotting. **L**–**N** Positive FJB signals in DG and EC in each group. Bar = 50 μm, *n* = 5/group. **O** Latency to the platform (Two-way ANOVA). **P** Frequency of platform crossings. **Q** Target quadrant time (%), and (**R**) Opposite quadrant time (%). **S** Representative tracking. **P < 0.01, and ***P < 0.001, all compared with controls (One-way ANOVA). ^#^*P* < 0.05, ^##^*P* < 0.01, and ^###^*P* < 0.001, compared with that in hypoxia+saline group (One-way ANOVA with Dunnett’s T3 post-hoc test).

Immunohistochemistry revealed significantly attenuated LC3B/TOMM20 levels in DM-treated mice than in saline-treated mice from 24 h after hypoxia treatment (Fig. 4A–H; Supplementary Fig. 2C, D). Additionally, western blotting confirmed reduced LC3B levels in DM-treated mice than in saline-treated mice at 24 h and 3 days after hypoxia (Supplementary Fig. 2A, B).

Accompanying the increased mitophagy levels, there were significantly lower activated caspase-3 levels at 24 h and 3 days (Fig. 4I, K) in DM-treated mice than in saline-treated mice. Meanwhile, caspase-3 levels were elevated (Fig. 4J, K). FJB staining results revealed significantly reduced neuronal damage in the hippocampus, PC, and EC due to DM treatment (e.g., DG, P < 0.001; EC, P < 0.001; Fig. 4L-N).

Cognitive abilities were evaluated by the Morris water maze at 1.5 months after hypoxia. Hypoxia administration caused cognitive defects. There was a prolonged latency to find the platform (*P* < 0.001, Fig. 4O), and an increase in opposite quadrant time (*P* < 0.001, Fig. 4R), with an accompanying reduction in target quadrant time (P < 0.001, Fig. 4Q), and number of platform crossings (*P* < 0.001, Fig. 4P). However, hypoxia-induced learning and memory defects were attenuated by DM treatment. For example, there was a reduced latency to find the platform (*P* < 0.001, Fig. 4O), an increase in number of platform crossings (*P* = 0.009, Fig. 4P), reduced percentage in the opposite quadrant time (*P* = 0.011, Fig. 4R), and increased percentage in the target quadrant time (*P* < 0.001, Fig. 4Q). Figure 4S shows representative space exploration trajectories.

### AOA treatment reversed succinate accumulation, oxidative stress, and iron stress induced by hypoxia treatment

**Fig. 5:**
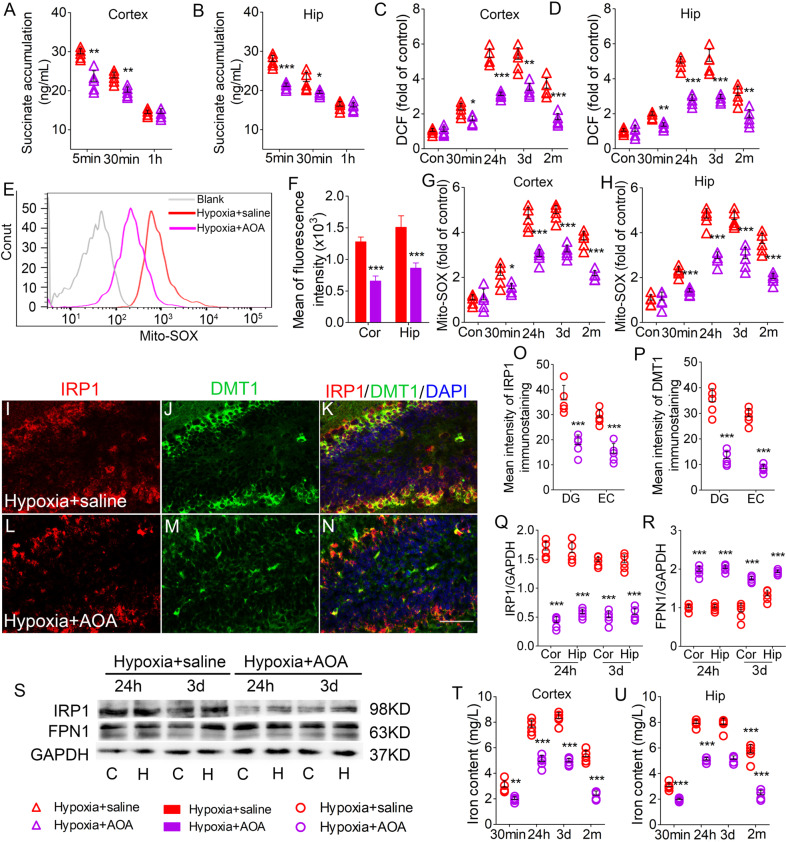
AOA reversed the succinate accumulation, oxidative stress, and iron stress induced by hypoxia treatment. Decreased levels of (**A**, **B**) succinate, (**C**, **D**) DCF level, and (**G**, **H**) mito-SOX in the hippocampus and cortex due to AOA treatment. **E**, **F** Flow cytometry-based quantification of hippocampal mito-SOX level. **I**–**N** Decreased immunoreactivity of IRP1 (red) and DMT1 (green) in the DG region after AOA treatment. Blue, DAPI. **O**, **P** Changed levels of IRP1 and DMT1 in the EC and DG. Bar = 50 μm. **Q**–**S** Expression of IRP1 and FPN1 in the cortex and hippocampus estimated using western blotting. *n* = 5/group. **T**, **U** Levels of iron (*n* = 5 per time point). *P < 0.05, **P < 0.01, and ***P < 0.001, all compared with hypoxia+saline group (One-way ANOVA).

In AOA-treated mice, there were significantly reduced levels of succinate (Fig. 5A, B), DCF (Fig. 5C, D), and mito-SOX (Fig. 5E–H) in the hippocampus and cortex. Figure 5I–P shows reduced DMT1 and IRP1 immunoreactivity in the DG and EC in AOA-treated mice. Immunohistochemistry detection revealed a similar reduction in other subregions of the hippocampus and PC (data not shown). Western blotting analysis confirmed reduced IRP1 levels (Fig. 5Q, S) and increased FPN1 levels (Fig. 5R, S) in AOA-treated mice. Additionally, AOA intervention reduced iron levels (Fig. 5T, U), which were increased after hypoxia treatment.

### AOA treatment reversed the hypoxia-induced increase in mitophagy, neuronal injury, and cognitive deficits

**Fig. 6:**
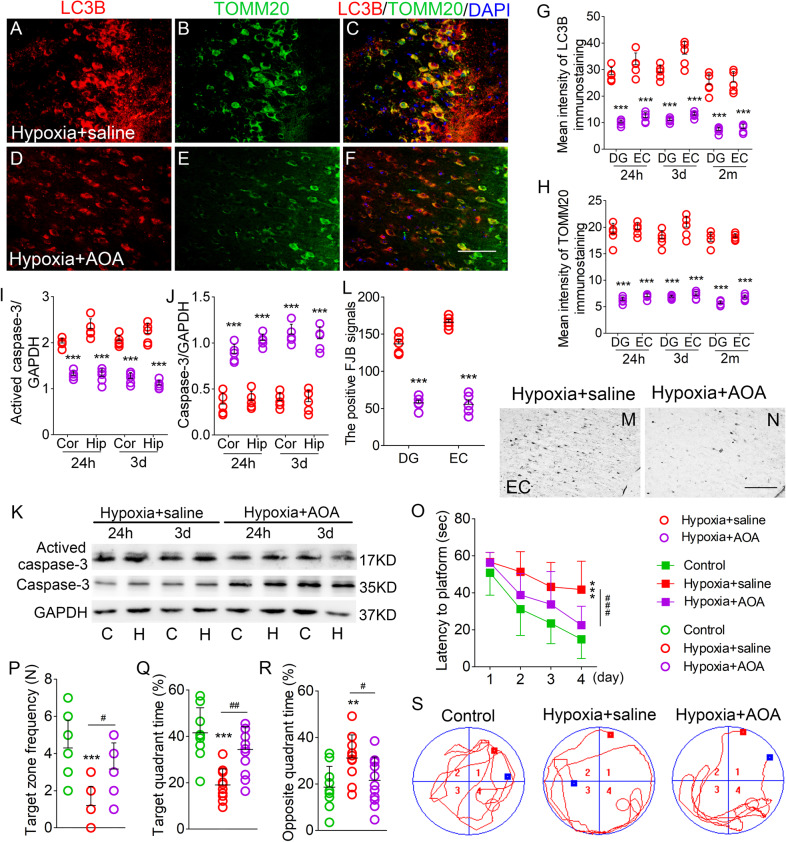
AOA reversed the hypoxia-induced increase in mitophagy, neuronal injury, learning and memory deficits. **A**–**F** Decreased immunoreactivity of LC3B (red) and TOMM20 (green) in the EC region after AOA treatment. Blue, DAPI. **G**, **H** Fluorescence intensity of LC3B and TOMM20 in DG and EC. **I**–**K** The expression of activated caspase-3 and caspase-3 in cortex and hippocampus detected by western blotting. **L**–**N** Positive FJB signals in DG and EC in each group after hypoxia. Bar = 50 μm. *n* = 5/group. **O** Latency to the platform (Two-way ANOVA). **P** Frequency of platform crossings. **Q** Target quadrant time (%). **R** Opposite quadrant time (%). **S** Representative tracking. **P < 0.01, and ***P < 0.001, all compared with controls (One-way ANOVA). ^#^*P* < 0.05, ^##^*P* < 0.01, and ^###^*P* < 0.001, compared with that in hypoxia+saline group (One-way ANOVA with Dunnett’s T3 post-hoc test).

AOA-treated mice showed significantly reduced LC3B/TOMM20 immunoreactivity in the cortex and hippocampus (e.g., DG and EC, Fig. 6A–H; Supplementary Fig. 3C, D). Western blotting results also confirmed the reduced LC3B levels (Supplementary Fig. 3A, B). Moreover, western blotting results revealed significantly decreased activated caspase-3 levels (Fig. 6I, K) and increased caspase-3 levels (Fig. 6J, K) after AOA treatment, which was accompanied by attenuated neuronal damage as shown by FJB staining (Fig. 6L–N). In the Morris water maze test, AOA-treated mice showed significantly improved learning and memory abilities than saline-treated mice (hypoxia + saline group; Fig. 6O–S).

### AICAR treatment reversed succinate accumulation, oxidative stress, and iron stress induced by hypoxia treatment

**Fig. 7:**
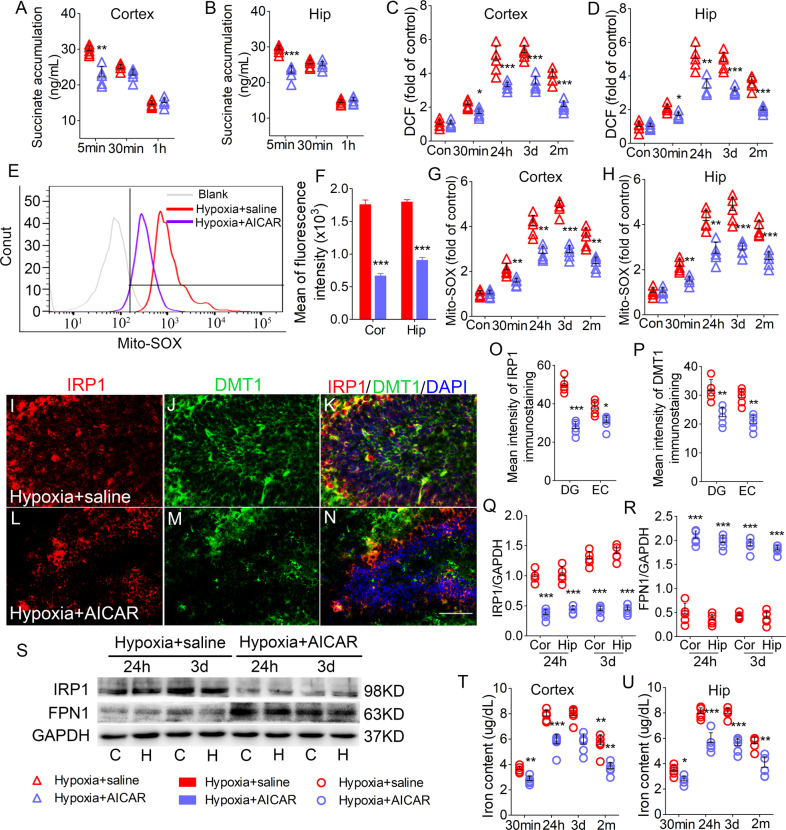
AICAR attenuated the succinate accumulation, oxidative stress, and iron stress induced by hypoxia treatment. Decreased levels of (**A**, **B**) succinate, (**C**, **D**) DCF, and (**G**, **H**) mito-SOX in the cortex and hippocampus due to AICAR treatment. **E**, **F** Flow cytometry-based quantification of hippocampal mito-SOX signal. **I**–**N** Decreased immunoreactivity of IRP1 (red) and DMT1 (green) in the DG region after AICAR treatment Blue, DAPI. **O**, **P** Quantified changes of IRP1 and DMT1 in the EC and DG. Bar = 50 μm. **Q**–**S** Expression of IRP1 and FPN1 in the hippocampus and the cortex estimated using western blotting. *n* = 5/group. **T**, **U** Levels of iron (*n* = 5 per time point). *P < 0.05, **P < 0.01, and ***P < 0.001, all compared with hypoxia + saline group (One-way ANOVA).

Similar to DM- and AOA-treated mice, compared with saline-treated mice (hypoxia + saline group), AICAR-treated mice showed significantly reduced levels of succinate (Fig. 7A and B), DCF (Fig. 7C and D), and mito-SOX (7E-H) in the hippocampus and cortex. Moreover, results of immunohistochemistry confirmed that AICAR treatment decreased immunoreactivity of DMT1 and IRP1 in the DG and EC (Fig. 7I-P), as well as in the other subregions of hippocampus and PC (data not shown). Additionally, western blotting analysis revealed that AICAR-treated mice showed decreased IRP1 levels (Fig. 7Q and S) and increased FPN1 levels (Fig. 7R and S). Additionally, AICAR-treated mice showed decreased iron levels than that in saline-treated mice (Fig. 7T and U).

### AICAR reversed the increased mitophagy levels, neuronal injury, and cognitive deficits induced by hypoxia treatment

**Fig. 8:**
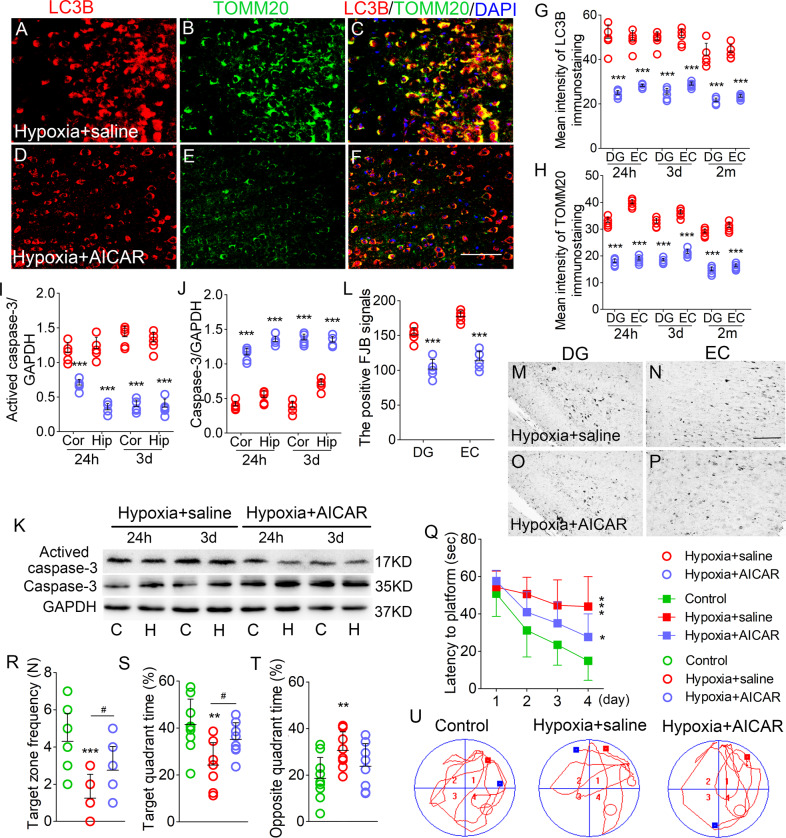
AICAR reversed the increased mitophagy level, neuronal injury, learning and memory deficits induced by hypoxia treatment. **A**–**F** Decreased immunoreactivity of LC3B (red) and TOMM20 (green) in the EC region after AICAR treatment. Blue, DAPI. **G**, **H** Quantification of LC3B and TOMM20 fluorescence intensity in DG and EC. **I**–**K** The expression of activated caspase-3 and caspase-3 in hippocampus and cortex detected by western blotting. **L**–**P** Positive FJB signals in DG and EC in each group. Bar = 50 μm, *n* = 5/group. **Q** Latency to the platform (Two-way ANOVA). **R** Frequency of platform crossing. **S** Target quadrant time (%). **T** Opposite quadrant time (%). **U** Representative tracking. *P < 0.05, **P < 0.01, and ***P < 0.001, all compared with controls (One-way ANOVA). ^#^*P* < 0.05, compared with that in the hypoxia+saline group (One-way ANOVA with Dunnett’s T3 post-hoc test)).

A significantly reduced fluorescence intensity of LC3B/TOMM20 in the hippocampus and cortex due to AICAR treatment was observed (e.g. DG and EC; Fig. 8A–H; Supplementary Fig. 4C, D). Western blotting analysis confirmed reduced LC3B levels in AICAR-treated mice compared to saline-treated mice (Supplementary Fig. 4A, B). Moreover, AICAR-treated mice showed decreased activated caspase-3 levels (Fig. 8I, K) and increased caspase-3 levels (Fig. 8J, K). Additionally, AICAR-treated mice showed significantly decreased FJB-positive signals than saline-treated mice (Fig. 8L–P). Further, AICAR treatment improved learning and memory impairment (Fig. 8Q–U).

## Discussion

This study investigated the pathogenesis of neonatal hypoxia and found that, in the early period after hypoxic insult, there were significantly increased succinate levels, iron levels, oxidative stress, with accompanying neuronal damage and cognitive defects. Inhibitors of SDH (DM), MAS (AOA), or PNC (AICAR) could attenuate succinate accumulation, while also attenuating the oxidative and iron stress, neuronal damage, and learning and memory defects caused by the hypoxic insult.

Neonatal hypoxia induces increased ROS production, which generates superoxide and further damages lipids and DNA, eventually causing neuronal damage by activating the calpain and caspase-3 pathways [40–43]. Additionally, ROS accumulation destroys intracellular Ca^2+^ homeostasis, and therefore regulates neuronal excitability and transmission, which makes neurons more prone to damage [43]. Excessive Ca^2+^ accumulation promotes mitochondrial dysfunction [44], which further increases ROS production [45] and promotes brain damage [46]. We previously confirmed that neonatal hypoxia induces oxidative stress, neuronal damage, and impaired cognitive function [2, 32].

Mitochondria are responsible for > 90% of ROS production [47–49]. Due to massive consumption of oxygen, mitochondria are susceptible to oxidative stress. A large amount of ROS is produced in damaged neurons due to hypoxia treatment and subsequently causes mitochondrial oxidative stress [2]. Conversely, the damage of mitochondria would lead to a decrease in mitochondrial membrane potential and produce a lot of ROS [47, 49, 50]. An increasing number evidence shows the relationship between mitochondrial oxidative stress and hypoxia-induced neuronal injury [51]. Consistent with previous reports, we found that neonatal hypoxia increased mitochondrial ROS levels and mitophagy, along with neuronal damage and cognitive dysfunction.

There is a direct dynamic relationship between oxidative stress and iron stress [52]. Excessive ROS promotes iron accumulation by activating IRP1 [26, 27]. IRP1 maintains intracellular iron homeostasis by regulating DMT1 and FPN1 [53]. IRP1 and DMT1 could be crucially involved in iron influx in the brain [54], and FPN1 is a vital iron export protein found in mammals [55, 56]. Additionally, the accumulated iron is involved in the Fenton reaction to produce excessive ROS, trigger oxidative stress, and ultimately cause neuronal damage [57, 58]. Consistent with previous reports, we observed altered levels of iron-related proteins and iron accumulation, which was accompanied by oxidative stress and neuronal injury. These results suggest a close relationship and synergetic contribution between oxidative stress and iron stress in neonatal hypoxia.

There is a close correspondence between succinate accumulation and ROS production [19, 21]. Physiologically, succinate is metabolized via the fumarate-malate-oxaloacetate pathway by SDH to maintain mitochondrial homeostasis [59, 60]. Under pathological conditions, reverse SDH catalysis causes succinate accumulation, which is then reoxidized and causes excessive mitochondrial ROS production [61, 62]. Therefore, reversal SDH catalysis is a vital source of succinate accumulation and mitochondrial ROS [17]. Fumarate, which is reverse catalyzed by SDH, is mainly produced through two main pathways [19]; namely, the MAS and PNC pathways [63–66]. Both pathways contribute to succinate accumulation under reverse SDH catalysis [61]. We found increased succinate levels in the early period after hypoxia treatment, which was followed by oxidative stress, iron accumulation, and neuronal injury. Reduced succinate accumulation attenuates oxidative and iron stress, accompanied by attenuated neuronal injury. This suggests that succinate accumulation in the early period after hypoxia is crucially involved in initiating oxidative stress, iron stress, and neuronal injury. Additionally, the inhibitors of SDH, MAS, or PNC reduced hypoxia-induced succinate accumulation. This suggests that hypoxia-induced succinate accumulation might mainly be due to reverse catalysis of SDH, which reversely catalyzing fumarate resourced from the PNC and MAS pathways.

NMDA receptor activation also contributes to succinate accumulation [21]. Neonatal hypoxia promotes glutamate release and NMDA receptor activation [10, 12]. Neonatal hypoxia induces succinate accumulation [23], which could further activate the NMDA receptor and eventually causes overexcitation-induced oxidative injury [21]. Additionally, NMDA receptor activation is involved in iron metabolism. Moreover, defects in excitotoxicity-induced iron ion metabolism cause iron stress and mitochondrial damage [67], which are the main sources of ROS production. Consequently, NMDA receptor activation may contribute to neuronal injury induced by succinate accumulation.

Epilepsy is a common brain disease, which ranks second after stroke in the nervous system [68, 69]. Epileptic seizures often occur in the neonatal period, with an incidence of 3–5% among live births [70]. Additionally, their most common cause is hypoxic-ischemic encephalopathy. Severe oxidative stress, with mitochondrial dysfunction and neuronal damage, is a vital characteristic of epilepsy [9]. Our findings confirmed that early succinate accumulation may be a critical initiator of neonatal hypoxia-induced oxidative stress and neuronal injury. Accordingly, succinate accumulation may contribute to neonatal hypoxia-induced epilepsy. This requires further experimental research.

In summary, we observed succinate accumulation in the early period after neonatal hypoxia administration. Moreover, succinate accumulation is crucial for initiating oxidative stress and iron stress. Reversing early succinate accumulation may attenuate oxidative stress and iron stress and mitigate neuronal damage and cognitive defects. This study provides new insight into the mechanisms underlying neonatal hypoxia and suggests that interrupting cumulative succinate is a potential therapeutic strategy.

## Supplementary information

Supplementary Figure Legends

Supplementary Figure 1

Supplementary Figure 2

Supplementary Figure 3

Supplementary Figure 4

## Data Availability

The data and material in this study are available from the corresponding author on reasonable request.
