# Supplementary material for: Attenuated succinate accumulation relieves neuronal injury induced by hypoxia in neonatal mice
Source: Cell Death Discov. 2022 Mar 28;8:138. doi: 10.1038/s41420-022-00940-7 (PMC8964675; doi:10.1038/s41420-022-00940-7)
Supplement: Supplementary file 1 — Supplementary Figure Legends [file 41420_2022_940_MOESM1_ESM.docx]

**Supplementary Figure 1**

Schematic of the experimental procedures.

**Supplementary Figure 2**

(**A, B**) The changed expression of LC3B in the cortex and hippocampus due to DM treatment was evaluated using western blotting. (**C, D**) Plots of pixel intensity of LC3B and TOMM20. ***P < 0.001, all compared with that in hypoxia+saline group (One-way ANOVA).

**Supplementary Figure 3**

(**A, B**) The changed expression of LC3B in the cortex and hippocampus due to AOA treatment was evaluated using western blotting. (**C, D**) Plots of pixel intensity of LC3B and TOMM20. **P < 0.01 and ***P < 0.001, all compared with that in hypoxia+saline group (One-way ANOVA).

**Supplementary Figure 4**

(**A, B**) The changed expression of LC3B in the cortex and hippocampus due to AICAR treatment was evaluated using western blotting. (**C, D**) Plots of pixel intensity of LC3B and TOMM20. ***P < 0.001, all compared with hypoxia+saline (One-way ANOVA).
