# Supplementary figures and images for: Attenuated succinate accumulation relieves neuronal injury induced by hypoxia in neonatal mice
Source: Cell Death Discov. 2022 Mar 28;8:138. doi: 10.1038/s41420-022-00940-7 (PMC8964675; doi:10.1038/s41420-022-00940-7)

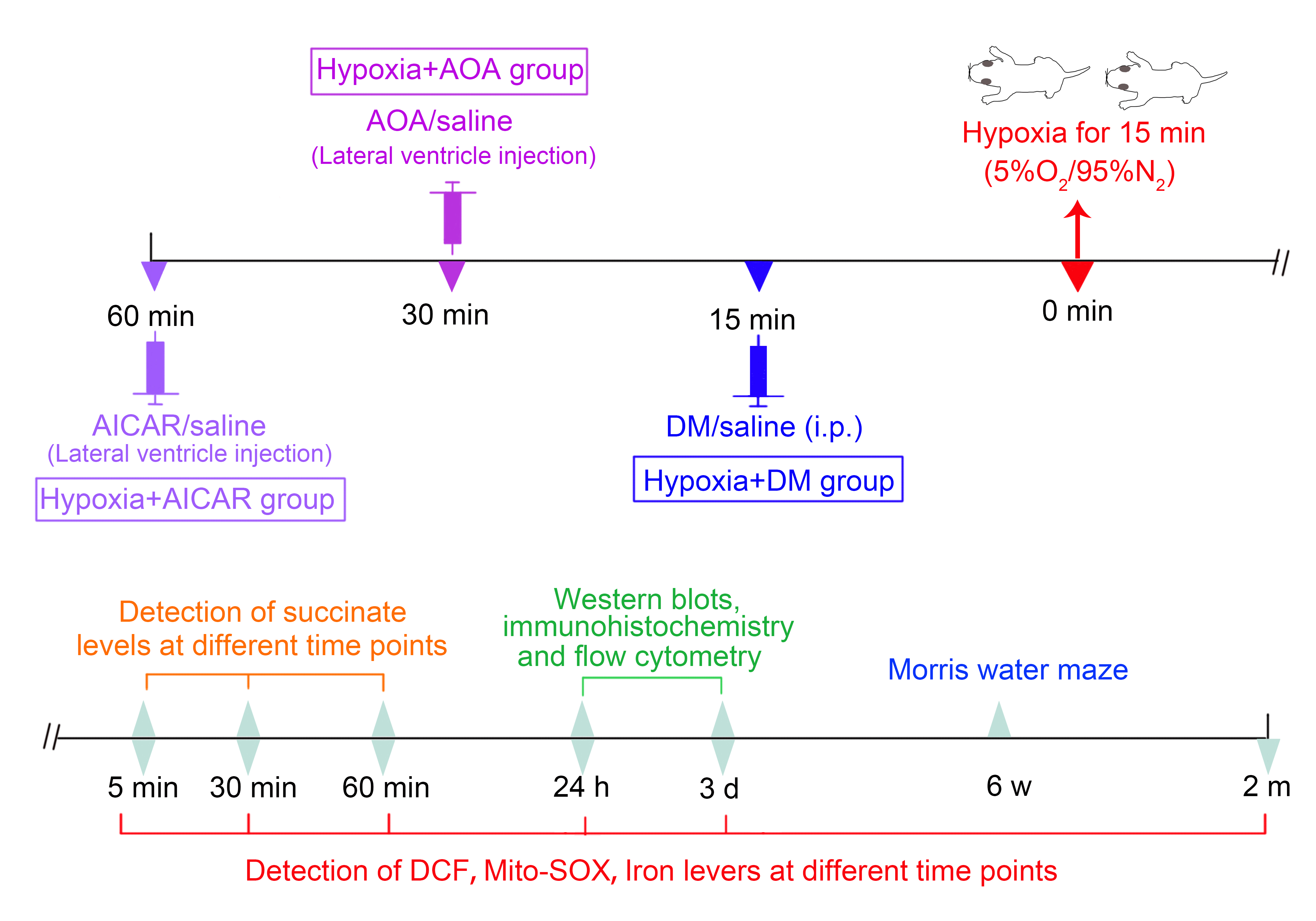

Supplement: Supplementary file 2 — Supplementary Figure 1 [file 41420_2022_940_MOESM2_ESM.jpg]

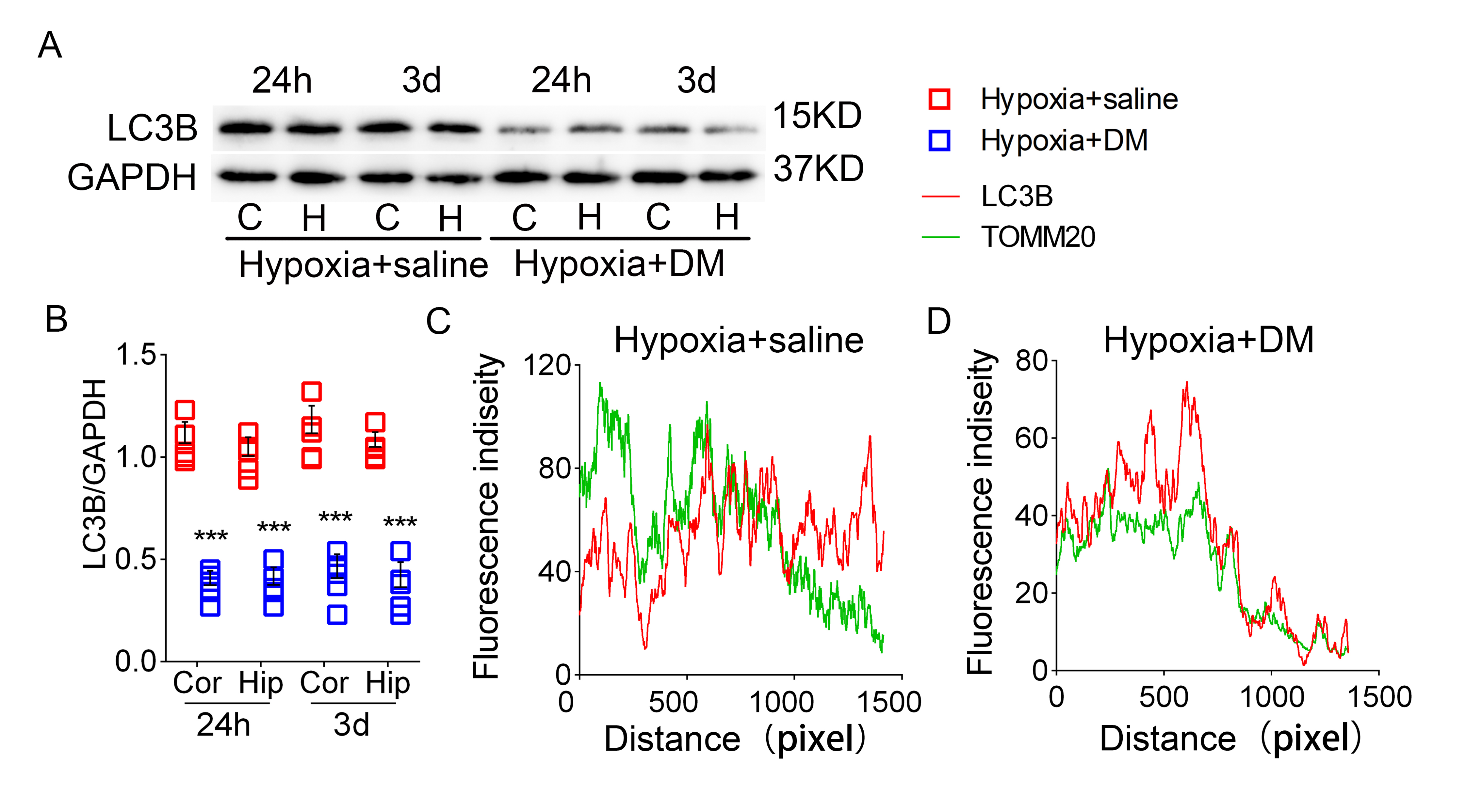

Supplement: Supplementary file 3 — Supplementary Figure 2 [file 41420_2022_940_MOESM3_ESM.jpg]

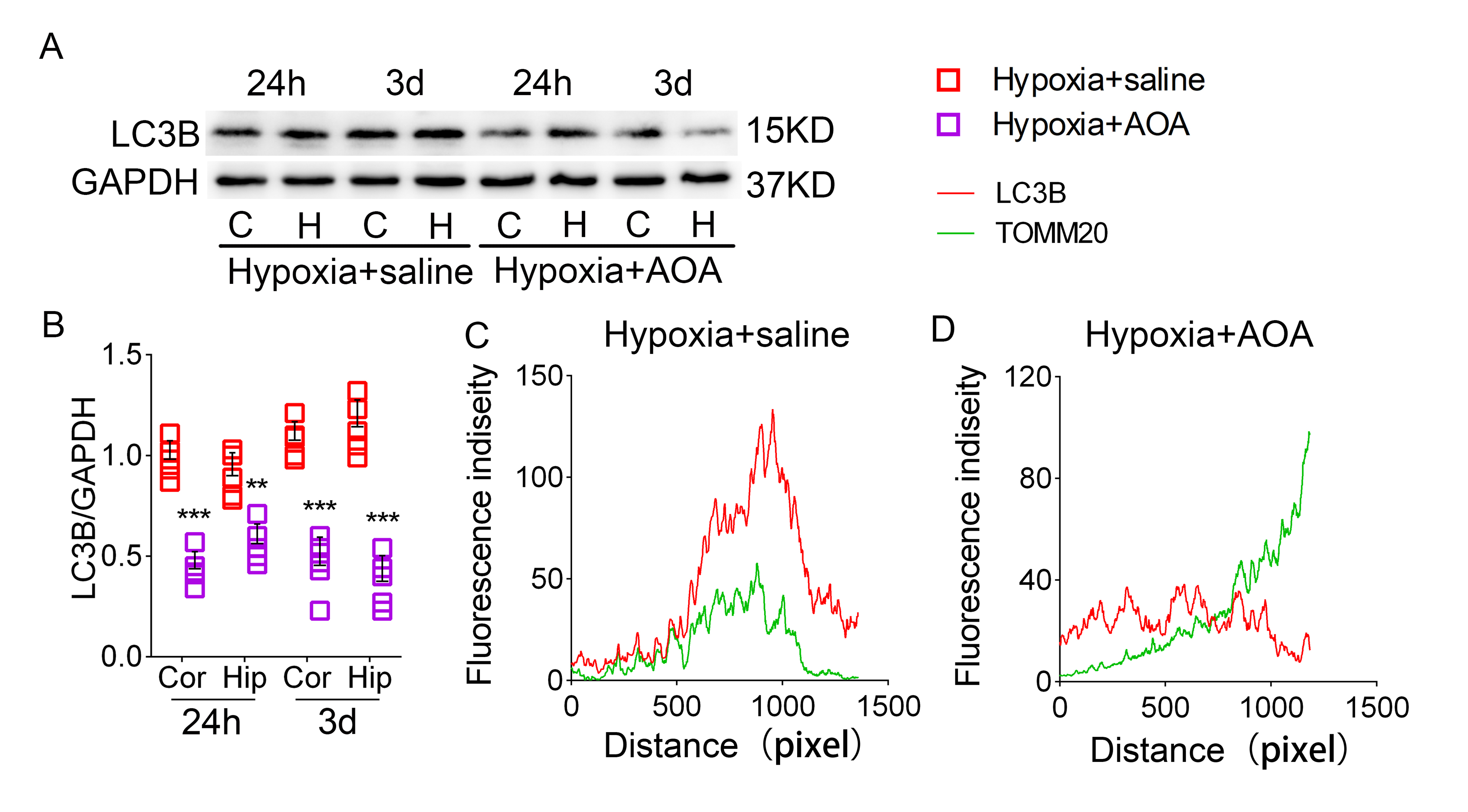

Supplement: Supplementary file 4 — Supplementary Figure 3 [file 41420_2022_940_MOESM4_ESM.jpg]

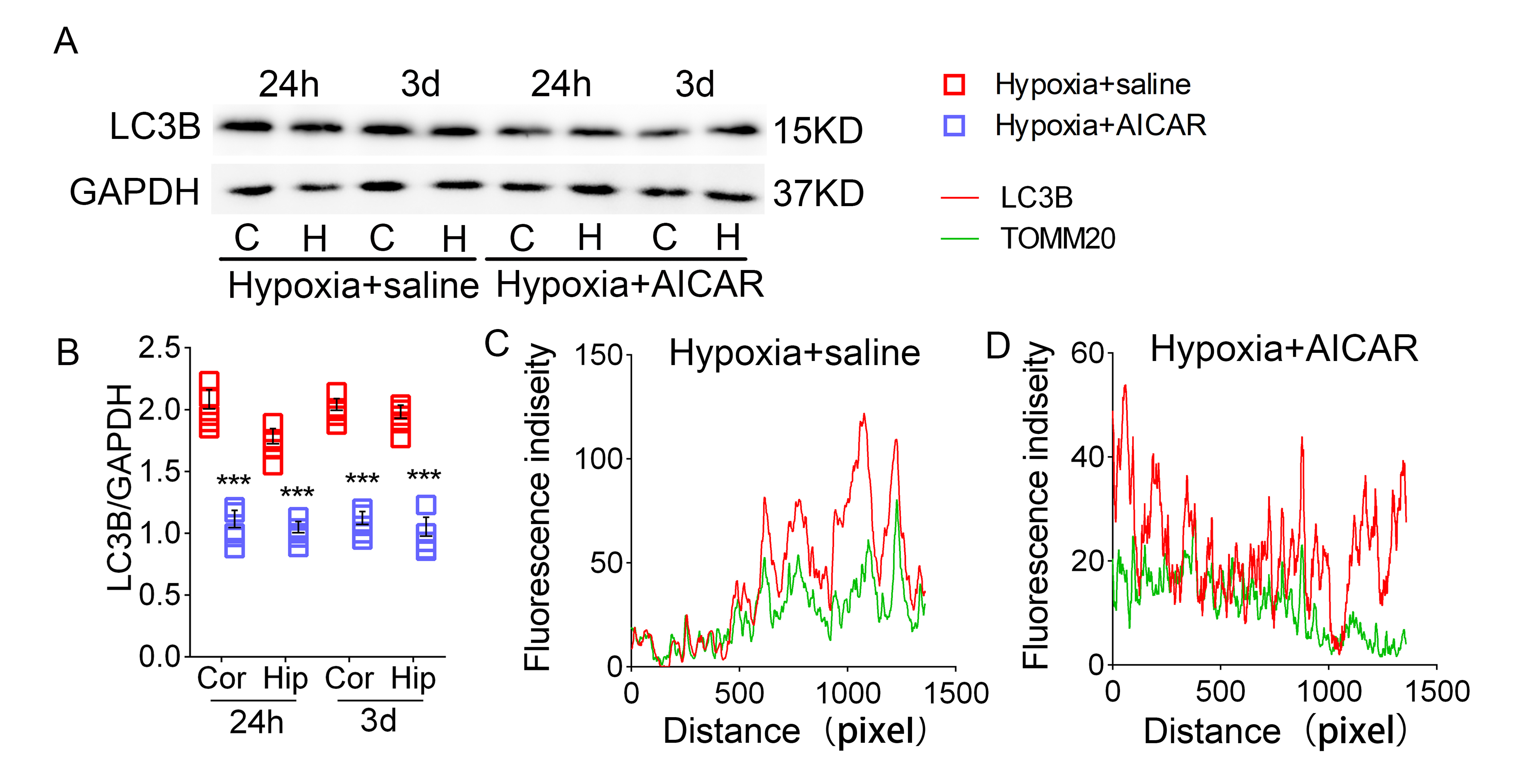

Supplement: Supplementary file 5 — Supplementary Figure 4 [file 41420_2022_940_MOESM5_ESM.jpg]
